# Difficulties facing healthcare workers in the era of AIDS treatment in Lesotho

**DOI:** 10.1080/17290376.2016.1179588

**Published:** 2016-04-29

**Authors:** Masebeo Veronica Koto, Pranitha Maharaj

**Affiliations:** ^a^ Masters in Population Studies, is a Researcher affiliated to the School of Built Environment and Development Studies at the University of KwaZulu-Natal, Durban, South Africa; ^b^ PhD, is a Professor affiliated to the School of Built Environment and Development Studies at the University of KwaZulu-Natal, Durban, South Africa

**Keywords:** difficulties, healthcare workers, AIDS, Lesotho, VIH, SIDA, Traitement, Professionnels de santé

## Abstract

Sub-Saharan Africa is most affected by the AIDS pandemic and Lesotho is no exception. In many countries, healthcare workers are at the forefront of the fight against AIDS. This study explores the difficulties facing healthcare workers in Lesotho using a combination of qualitative methods – focus group discussions and in-depth interviews. The findings suggest that healthcare workers are afraid of contracting HIV from their patients and this affects their delivery of services. In addition, the results revealed that poor infrastructure and shortage of supplies at the facilities hinder healthcare workers from performing their duties effectively. The other concern was the heavy workload and severe time constraints which puts enormous stress on healthcare workers. Stigma and discrimination emerged as major problems for healthcare workers. Addressing the challenges facing healthcare workers is essential in effectively managing the AIDS pandemic facing the continent.

## Résumé

L’Afrique subsaharienne est la région la plus touchée par la pandémie du SIDA et le Lesotho ne fait pas exception. Dans de nombreux pays, les professionnels de santé sont à la pointe de la lutte contre le SIDA. Cette étude utilise une combinaison des méthodes qualitatives telles que des discussions des groupes et des interviews en profondeur pour examiner les difficultés que rencontrent les professionnels de santé au Lesotho. Les résultats suggèrent que les professionnels de santé ont peur de contracter le VIH de leurs malades et ceci affecte leur prestation de services. En plus, les résultats ont révélé que l’insuffisance des infrastructures et le manque de fournitures dans des centres médicaux empêchent les professionnels de santé de travailler efficacement. L’autre préoccupation est la lourde charge de travail et des sévères contraintes horaires qui mettent une pression énorme sur les professionnels de santé. La stigmatisation et la discrimination ont émergé comme principaux problèmes pour les professionnels de santé. Relever les défis auxquels sont confrontés les professionnels de santé demeure un point essentiel dans la gestion de la pandémie du SIDA sur le continent.

## Introduction

Lesotho is a small, independent Kingdom in Southern Africa, surrounded completely by the Republic of South Africa. It has a total surface area of 30355 km^2^ and an estimated population of approximately 2 million people (Belle, Ferriera & Jordaan 2013). The country is roughly divided into four geographical zones: the mountain region, the lowlands, the Senqu River Valley and the foothills. At present, the country is ranked 158 out of 186 countries on the human development index (UNDP 2013). The economy is dependent on subsistence agriculture, manufacturing and the exportation of labour to neighbouring South Africa. Poverty is widespread throughout the country, with 43.4% of the population living on less than $1.25 a day (UNDP 2013). The country has been severely affected by the AIDS pandemic and has the third highest HIV prevalence rates in the world. Since the first case of HIV was identified in 1986 the number of new cases has steadily increased. By 2005, there were about 270,000, people living with HIV in Lesotho and this number increased to 360,000 by 2013 (UNAIDS 2006, 2014). In 2013, the adult HIV prevalence rate in Lesotho was 22.5% (UNAIDS 2014). Despite the relatively late arrival of AIDS in Lesotho, HIV has been spreading rapidly throughout the country, affecting both rural and urban areas alike (Joseph, Rigodon, Cancedda, Haidar, Lesia, Ramangoaela, *et al*. 2012). AIDS is having a devastating impact on the country, undermining many development gains. It is the main cause of mortality, accounting for 56% of deaths among children under five, and is responsible for the decline in life expectancy (Cohen, Lynch, Bygrave, Eggers, Vlahakis, Hilderbrand, *et al*. 2009). The AIDS pandemic has negatively affected a number of sectors but the health sector is the one that is directly affected due to the large number of people seeking care, high patients’ healthcare costs and demand for more healthcare workers (International Labor Office 2005). Like many other parts of Africa, Lesotho is also facing a severe shortage of healthcare workers. This is supported by Joseph *et al*. (2012) who notes that about 60% of professional nursing posts are vacant and there are fewer than 100 (0.5 physicians per 10,000 population) physicians working in the country. A number of studies attribute the shortage of healthcare workers in Lesotho to migration to the USA, Saudi Arabia and other countries (Joseph *et al*. 2012; Ntlale & Duma 2012). The nature of the AIDS pandemic in Lesotho raises salient concerns about the extent to which the health system is responding to the increasing demands for health care in the context of a severe shortage of healthcare workers.

In their paper, Case and Paxson (2009) document the impact of the AIDS crisis on non-HIV-related health services in 14 sub-Saharan African countries using data from multiple waves of Demographic and Health Surveys (DHS) for each country. Their analysis reveals deterioration in the delivery of health care which was highly correlated with an increase in AIDS prevalence. They conclude that the decline in the quality of health services could be possibly due to ‘the redirection of funds for supplies and trained medical professionals to care for HIV patients, or perhaps to a reduction in the supply of trained personnel as a consequence of the pandemic’ (Case & Paxson 2009). The AIDS epidemic has multiplied the burden of disease and as a result, demands for health care. As the level of HIV infections increases, more and more people present at health facilities with opportunistic infections that attack the immune system. The increasing availability of antiretrovirals has also led to an ever increasing demand for health services. Providing lifelong AIDS treatment requires additional health workers (Naicker, Plange-Rhule, Tutt & Eastwood 2009). As a result, the demand for and cost of public healthcare services has increased, crowding out other health conditions and doubling bed occupancy rates in a number of African countries (Steinberg, Johnson, Schierhout, Ndegwa, Hall, Russell, *et al*. 2002; Tamiru & Haidar 2010).

A review of the literature suggest that the general picture of the health sector impacted by HIV/AIDS in developing countries is one of changing disease patterns requiring greater skill levels and diagnostic facilities, rising demands for beds, increased costs and a demoralized and overburdened workforce (Tawfic & Kinoti 2006). The extent of the spread of HIV infections in Sub-Saharan Africa has meant that many people are affected by either an AIDS death or illness. Healthcare workers themselves not only confront AIDS at their workplace but also are affected due to their sick families, friends, neighbours and colleagues. When they finish work at the end of the day they are faced with those that are terribly ill at home and it becomes hard for them to disengage themselves from their profession while they are at home (UNAIDS 2000). Healthcare workers face additional occupational risks from accidental exposure to blood or blood products or handling non-sterile injecting equipment (Tawfic & Kinoti 2006). Some of the practices that aggravate the transmission of HIV/AIDS in the healthcare settings are the high demand of unnecessary injections that are being administered, the use of non-sterile needles when supplies are low and the disposal of hazardous waste that is not properly regulated by health facilities (Board 2001). In addition, healthcare workers may have a heightened risk of HIV infection because of their own sexual behaviour. A study conducted in Zambia to assess HIV/AIDS risk-taking and status awareness found that almost one quarter of sexually active health workers had multiple partners and more than a third had not used condoms (Kiragu, Ngulube, Nyumbe, Njobvu, Eerens & Mwaba 2007).

Healthcare workers are the first point of contact for ill people regardless of type of disease (Jennings 1990). They are essential in the prevention and management of disease in all areas of the population (United Nations 2003). This study builds on previous research but looks specifically at the experiences of healthcare workers in the era of AIDS treatment. Among the numerous studies that have been conducted in Lesotho specifically with regard to the AIDS pandemic, there has been relatively limited focus on understanding the difficulties facing this sector of the population. To address this gap, the focus of this study is on the perspectives and experiences of healthcare workers in the era of AIDS treatment.

## Methods

The study draws on mixed, qualitative data derived from in-depth interviews and focus group discussions with healthcare workers. The study was conducted with healthcare workers at three health facilities in the district of Maseru, which is the capital of Lesotho. There are about 40 healthcare facilities in this district which are government owned, church owned, some owned by non-governmental organizations and others privately owned. The study was restricted to health facilities that are directly providing services to men and women living with HIV and AIDS. The objective of the in-depth interviews and focus group discussions was to gain detailed insights into the difficulties facing healthcare workers by exploring how the epidemic has affected their work environment. It also looked at how they are coping in the context of a shortage of healthcare workers.

The study draws on in-depth interviews and focus group discussions. A total of 10 in-depth interviews were held with healthcare workers. The majority of the healthcare workers interviewed were female and registered nurses. Only two interviews were held with male healthcare workers. The study relies on a relatively small sample but this is not a problem given that the main focus of the study is not generalizability but to better understand the perspectives and experiences of healthcare workers. This was largely an exploratory study aimed at getting intricate personal views and perspectives on lived experiences and feelings of healthcare workers. Focus group discussions were also conducted with the healthcare workers to complement the in-depth interviews. For this study, two focus group discussions were conducted. The focus group discussions consisted of four to six female healthcare workers. The primary intention of holding focus group discussions was to get beneath the surface of a particular topic, presuming that individuals will reveal more when they are stimulated by the camaraderie and comments of others.

The use of mixed, qualitative methods allowed for exploration of a range of views and experiences. Due to time constraints in health facilities, nonprobability sampling techniques were used to recruit healthcare workers. For the in-depth interviews and focus group discussions, non-probability, convenience sampling was used to select healthcare workers for the study. Healthcare workers were approached directly and asked if they would be willing to participate in the study and to talk about aspects of their work lives. In addition, they were asked to complete an informed consent form. The researchers assured the participants that confidentiality, anonymity and privacy would be strictly observed. Both the focus group discussions and in-depth interviews were tape recorded with the permission of the healthcare workers. The interviews were conducted in the local language (Sesotho). The in-depth interview lasted approximately 45 minutes and the focus group discussion approximately one and a half hour. Ethical clearance for this study was obtained from the University of KwaZulu Natal and the Ministry of Health and Social Welfare in Lesotho.

## Data analysis

The tapes were transcribed and translated into English. All the transcribed interviews were read and reread and organized according to major themes and assigned initial codes. Initial coding refers to the process of breaking down, examining, comparing, conceptualizing and categorizing data (Punch 2005). After initial coding, all the data were assembled under major themes. In the final analysis, the codes were modified and recurrent themes that emerged across the transcripts were identified. The aim was two-fold: firstly, to identify common themes and secondly, to identify issues of controversy and debate. The transcripts are used to illustrate particular findings from the in-depth interviews and focus group discussions.

## Results

Perception of risk of HIV infection is high among healthcare workers. Worries about personal safety are one of the most pervasive and enduring issues influencing perception of risk of HIV infection among healthcare workers. Their worries stem from the high volume of AIDS patients at their health facility and their inability to sometimes identify infected persons. In addition, some of patients they cared for presented later with advanced symptoms. In the in-depth interview one healthcare worker explained:

There are patients that one avoids to serve because it is evident by just looking at them that they are HIV positive or even have full blow-AIDS and seem very ill. You are even scared to touch them and the scary part is that we have to take a needle and inject them. However, I have to take their blood pressure, weight and sometimes ask them to take their clothes off. Then you find out that they even have a rash or open wound on their bodies and they normally do not mention when you talked to them about their problems. Normally, I become so scared and frustrated when I come across cases like that one. Sometimes you just have to pray that God protects you from getting this disease while helping patients. (IDI # 8)

In the interviews, healthcare workers noted that they are accustomed to treating HIV infected patients because of their training but they still have fears about their own risk of HIV infection. High perceived risk of HIV infection may influence their ability to carry out their duties effectively. Healthcare workers are particularly nervous about conducting physical examinations on some patients because they are critically ill and have open wounds. They indicated that sometimes they are tempted to avoid some of their patients by referring them to their colleagues. However, they are aware of their professional responsibilities, and that they have sworn an oath to take care of all their patients.

The shortage of supplies such as protective equipment also put healthcare workers at risk of contracting HIV. Healthcare workers may sometimes perform clinical duties with HIV positive patients without wearing gloves. The shortage of supplies makes it difficult for them to protect themselves. Sometimes their suppliers run out of stock or the health facilities themselves run out of stock because the order was not placed on time and the delivery of supplies is done when they already do not have anything to use. Although healthcare workers had received training in managing AIDS patients they were still concerned about coming into contact with contaminated blood, especially as a result of shortage of basic protective equipment. They reported performing clinical duties without wearing gloves and blamed the system for not providing them with all the necessary equipment needed for protecting them from infectious diseases. There is some concern about accidental needle stick injury; even though studies suggest that the risk of HIV infection is low. The fear of HIV infection is revealed in the following comment by a nursing assistant:

Yes, we inject people and sometimes you miss the target or a patient jumps and you accidentally prick yourself with the needle that injected an HIV positive patient. The majority of the patients I serve are HIV positive. I am always scared that I may prick myself and become infected. I think sometimes I will even make a mistake because I always think about what I would go through or what I would do if I was HIV positive. (IDI #1)

Healthcare workers observed that they frequently come into contact with contaminated blood and this heightens their risk of HIV infection. One midwife stated:

When you help a woman to deliver you are getting in contact with the blood of the mother. When the baby comes out, blood just splashes and obviously, when you catch the baby blood comes at you. You would be very lucky if you can manage to avoid it. Sometimes when a woman delivers and encounter problems, you as a nurse have to assist and pull the baby out. In the process, you get in contact with blood even if you are wearing gloves. You will find that gloves are too small to cover your arms. The number of women who give birth per day in this facility is very high and there is not enough staff. There are times when we have to conduct delivery after delivery. There are only three of us in the delivery ward and sometimes more than three women need to be attended to at the same time. In cases like this, we are not even able to look for or put on the gloves. We just aim at saving both the baby’s and the mother’s lives. (IDI #2)

The situation at health facilities is very stressful for healthcare workers. As the level of HIV infections rises the demand for health care also increases. The shortage of healthcare workers is one of the main problems facing the country. There are many vacant positions that are difficult to fill. This adds to the work burden on the remaining health professionals. In addition, high absenteeism is aggravating the shortage of staff in health facilities. If a staff member is absent from work, this leaves a big gap for others to fill since there is already a shortage of human resources. There is a long list of tasks that healthcare professionals are charged with on daily basis and they cannot afford to perform all these tasks. The shortage of staff has meant that they had to assume additional responsibilities and they do not have the option of refusing to undertake certain tasks. In addition, they reported feeling discouraged when they see some of their patients becoming progressively worse over time. They feel that they are working extremely hard to bring about positive changes in people’s lives; however they feel they are not winning the battle against AIDS because of the high volume of patients. The combination of long working hours and a high client load puts healthcare workers under a great deal of pressure. They also find it difficult to juggle their professional and personal life. Many of the healthcare workers stated that they find themselves confronted with competing demands and they are struggling to cope. Some healthcare workers feel guilty about working long hours at the health facility. This is clear in the following comment:

The workload in this facility is killing us. I do not know whether in other facilities there is still the same problem of working like this. There are only two of us in this ward today. Sometimes you find that you are alone in the ward and you have to help patients, give them medication and maybe help them to change. You may need someone to help turn the patient and there is no one. Sometimes we cannot even identify emergency cases or patients that need immediate attention. When I get home, I feel so tired that I cannot even cook for my family. I am relying on the domestic worker to take care of my family and this does not satisfy me at all. (IDI #6)

The infrastructure also does not allow the healthcare workers to perform their duties effectively. At the time when the buildings were constructed no provision was made for some of the activities that are currently performed at the facilities. The interviews suggest that there is a need for more consulting rooms and other facilities to allow for greater privacy and confidentiality. In addition, inadequate infrastructure may ultimately result in long queues that will increase the workload of health workers. As much as the health sector is working hard to make services more accessible there is a need to build more structures for these services to run properly. One healthcare worker explained in the focus group discussion:

There are situations where one counselling or examination room is shared by more than one health care worker when giving one to one counselling sessions to clients. In other situations, counselling rooms are separated either by a curtain or a thin board that is not sound proof. The health care workers in all these cases wish that they can do something to protect their patients but there is nothing they can do. All these situations compromise confidentiality and privacy. However, the alternative would be serving patients one at a time which would result in long queues and a long waiting period for patients. This would also result in some patients having to go back home without having been consulted. (FGD #2)

The amount of work done by healthcare workers is quite labour intensive and the number of professionals assigned to perform the tasks is very small. Healthcare workers complained about the need for more staff to provide services efficiently and effectively. They explained that their workload is quite large and the activities that they need to complete are usually complex and time-consuming. As a result, they are always physically exhausted and suffering from high levels of stress. They feel that their working conditions are also negatively impacting on their family life. One healthcare worker elaborated on her working conditions:

There is this thing called burnout syndrome. I think we, health workers are suffering from that. We are always tired and we cannot even perform our duties as expected. Normally when I wake up in the morning, I already feel tired. Sometimes I just feel in a bad mood without knowing what happened. You just find yourself discouraged and down. We need attention as well just like our patients. We counsel our patients but we do not have anywhere to be counselled. This causes a strain on us. We cannot even counsel each other because there is a lot of work and we are all tired. Our problems are our families. This strain spills over to our families because when we get home we talk about work all the time. We do not discuss family matters that need to be solved. (IDI #3)

Healthcare workers also pointed out that sometimes their stress at work spills over to their families as they take their patients’ problems along with them when they go home. They are always concerned about their patients and their problems and how they are suffering. Healthcare workers also stated that sometimes they get discouraged when they see the condition of their patients deteriorating when they come for check-ups at their next visit or when they come to collect their medication. They are expecting to see their patients getting better and go on with their lives instead some of them become worse.

Healthcare workers reported that they feel sorry for some of their patients that are critically ill and have no one to take care of them at their homes. They also feel sad for their patients because there is no cure for AIDS even though treatment is available. They indicated that they become attached to their patients and feel like the patients are part of their families. This is because they spend a great deal of their working lives interacting closely with AIDS patients. In the in-depth interviews healthcare workers explained the difficulty of detaching themselves from their work. This is revealed in the following statement: ‘you cannot stop thinking about them even when you are at home. Some of them tell me their sad stories and backgrounds and I feel so sorry for them so much that I become attached to them’ (IDI #4). This suggests that healthcare workers develop a strong bond with their patients. They often find themselves becoming emotionally attached to their patients. One healthcare worker recalled a case of an older woman who was HIV positive and was on antiretroviral treatment. She said:

There is this particular patient and she is a regular client because she is on antiretroviral treatment. She is an old woman who is in her sixties. She told me when she started her treatment that she was taking care of her granddaughter’s little child and she did not have any idea that the child was HIV infected. The child got ill and died after a while. This woman became sick six month after the baby died. She was advised to take an HIV test when she did not get better and the test came out positive. She told me that she is so afraid that no one is going to take care of her when she gets ill because her granddaughter is also HIV infected. When somebody tells you their problems like this, personally you feel like you should go in and help. (IDI #6)

Healthcare workers reported that they are not supposed to provide handouts of any form to patients because it could cause some problems if others are not given anything but sometimes they offer some money or second hand clothing to some of their patients. They feel unable to turn a blind eye to the problems that their patients are experiencing in their daily lives. The majority of patients visiting the health facilities in this research are reported to be poor and in need of assistance in the form of food or finances particularly for transport. Healthcare workers are concerned that their patients do not have proper nutrition and they sometimes take medication on an empty stomach. Many often do not have money to travel to the health facility for their treatment

Healthcare workers reported that they themselves were reluctant to go for a HIV test. They are afraid that if they are found to be HIV positive, they would be seen as having behaved badly. The association of AIDS with sexual promiscuity is still prevalent in the community and healthcare workers are not the exception in this regard. Fear of stigma and discrimination also results in healthcare workers delaying testing until they are very sick and already have full-blown AIDS. There remains some silence and secrecy surrounding this disease, even among health professionals. Healthcare workers are also afraid of disclosing their HIV status because of fear of negative comeback. They are particularly worried about being the subject of gossip. One healthcare worker said:

You will be surprised that we as health care professionals do such things as gossiping about each other. If one of our colleagues shows some HIV related symptoms such as weight loss, or being suspected of having tuberculosis other colleagues start talking behind his or her back. As a person, you realize that even if I have had HIV I would not disclose my status in this place. This is not the right place for me to talk about something like that. (IDI #4)

Fear of stigma and discrimination are also challenges that obstruct healthcare workers from knowing their status.

## Discussion

This study was conducted in a setting where the demand for health services is particularly strong given the high HIV prevalence. The results of this study are of special interest because healthcare workers are at the forefront in the fight against the AIDS pandemic. Generalization of results to the whole country must be cautious because of the limited geographical scope of the study and the small sample size. However, this study sheds insights into the perspectives and experiences of healthcare workers in a high HIV prevalence setting.

In the study, personal safety among the healthcare professionals was an important issue. Healthcare workers are afraid of becoming infected at their place of work. Accidental needle stick was reported as one of the possible ways that healthcare workers can become infected. Estimates suggested by prospective studies indicate that on average the risk of HIV transmission after a percutaneous exposure to the blood that is infected by HIV is approximately 0.3 percent and that after mucous membrane exposure is 0.09 percent (Sadoh, Sadoh, Fawole, Oladimeji & Sotiloye 2009). Even though research suggests that the chances of contracting HIV through needle-stick are small (Parker, Aggleton, Attawell, Pulerwitz & Brown 2002), healthcare workers probably feel that the possibility that it might occur is high due to the large number of HIV infected people that are being cared for at health facilities. The study revealed that the fear of HIV contagion among the healthcare workers caused them a great deal of worry. However, it is also leading to poor service delivery because some patients are being avoided by healthcare workers because of their HIV positive status.

Inadequate training in clinical management of HIV is a major problem for healthcare workers. Limited knowledge of HIV/AIDS management may lead to healthcare workers getting infected at work and/or carrying out medical interventions that are inappropriate (Evans & Abiteboul 1999). Other studies suggest that clinical management skills of nurses are improved through out-of-facility intensive training and in service mentorship (Cohen *et al*. 2009). In their study, Cohen *et al*. (2009) observed that the training of nurses has led to an improvement in their diagnostic and management skills which in turn assisted in boosting morale and confidence in providing HIV/AIDS care and treatment. The existing infrastructure also does not allow the healthcare workers to carry out their job properly, particularly when it comes to counselling and consultation. These procedures need to be performed in a private place to ensure maximum confidentiality. However, some of the consulting rooms at the facilities are divided by curtains or thin boards that are not sound proof. This compromises confidentiality as it is possible that other people might overhear what is being discussed in the consultation room. There is therefore a need for substantial infrastructure improvements to absorb the increasing number of patients visiting health facilities. In their study, Cohen *et al*. (2009) noted that this may involve providing essential equipment, improving organizational capacity with basic furniture and supplies, improving conditions as well as improving infection control and occupational health practices.

Stigma and discrimination are very powerful in preventing people from knowing their HIV status. Many people consider HIV/AIDS as a disease that affects ‘others’ (Melby, Boore & Murray 1992). There remains a commonly held belief that only certain groups of people are at risk of HIV infection. Examples include marginalized groups of people such as sex workers and men who have sex with other men. Stigma and discrimination compels individuals to hide their HIV status and this causes the epidemic to spread further. Healthcare workers like the rest of the population fear that they will become the focus of stigma and discrimination by their colleagues as well as the broader community. Gossip and rumours that emerge discourages those who would want to test for HIV from taking the test. Furthermore, healthcare workers are concerned that if their HIV status was revealed then their family members will suffer because they will be treated badly by other members of the community. For all these aforementioned reasons healthcare workers may prefer to hide their status. However, hiding one’s HIV status can lead to further stress, social isolation and depression (Melby *et al*. 1992; Petros, Airhihenbuwa, Simbayi, Ramlagan & Brown 2006).

In the interviews healthcare workers reported feeling highly stressed. There are a number of factors contributing to the healthcare workers prolonged stress, which ultimately leads to burnout. The number of patients seeking healthcare services has increased and the healthcare workers have to work extra hours in a day so that they can at least attend to as many people as possible. Jennings asserts that ‘the nurse’s role has long been regarded as stress-filled based upon the physical labour, human suffering, work hours, staffing and interpersonal relationships that are central to the work nurses do’ (Petros *et al*. 2006). Therefore, the number of hours that healthcare workers have to work per day negatively affects them. In some health facilities, healthcare workers are not able to serve all the patients waiting for services in a day. They have to send some of them back and request that they return the following day. Some of the patients never come back to the facility because their condition will have deteriorated or they will have died. Studies suggest that patient outcome is one of the acute factors that contribute to healthcare worker stress (Petros *et al*. 2006). If the health professional finds out that after sending patients home without attending to them they died or are not able to walk to the facility again, they get frustrated and start blaming themselves for failing to assist their patients.

Shortage of qualified healthcare workers is one of the disturbing issues facing developing countries and it is likely to be a critical impediment to the achievement of the Millennium Development Goals. The shortage of healthcare personnel is exacerbated amongst other things by the emigration of skilled healthcare professionals to other countries. Absenteeism is common among healthcare workers. Due to the increased number of patients seen daily, healthcare workers find themselves increasingly under stress. They also fall sick from AIDS-related illnesses. These reasons contribute a great deal to the shortage of staff at health facilities. In their review, Ntlale and Duma (2012) identify a number of factors that forces nurses to leave their home countries. They note that inadequate remuneration and the inability to save money as well as poor working conditions are among the factors that force nurses to leave their home countries. In Lesotho, the Ministry of Health has partnered with Irish Aid and the Clinton Foundation to alleviate some of the shortages in staff at health facilities. They have been actively involved in initiatives to recruit health professionals from other African countries including Zimbabwe and Kenya. A Review of Irish Aid – Clinton Partnership (2010) observed that in total 150 nurses of Basotho (40), Kenyan (60), and Zimbabwean (50) nationalities were hired and placed at health facilities across the country. As a result patients across the country were able to visit health facilities close to their homes and this led to a reduction in the distances they have to travel to access health services. The partnership offered incentives to the nurses by improving their living conditions – they renovated sites with inadequate housing, as well as provided the nurses with appropriate furnishings. In addition, those nurses employed in remote parts of the country were given a hardship payment known as a mountain allowance. The partnership was able to attract health professionals to work in rural areas in Lesotho by offering them initiatives. There should be more initiatives like this to try and improve the living and working conditions of healthcare workers. They also need to feel like they are being valued. Migration of healthcare workers from developing countries is likely to lead to poor health service delivery and in some instances, the complete collapse of healthcare systems (Ntlale & Duma 2012).

Innovations such as task shifting can also be applied to deal with the shortage of healthcare workers. Task shifting refers to the transferring of responsibilities. According to Joseph *et al*. (2012:144), task shifting has been defined as ‘the delegation of tasks from higher qualified to lower qualified cadres’. In 2006, a community health worker (CHW)-based project called ‘The Rural Initiative’ began in the remote mountain regions of the country (Rigodon, Joseph, Keshavjee, Cancedda, Haidar, Lesia, *et al*. 2012). The project involved recruiting and training community health workers from the local populations. The community health workers were involved in a number of activities, including community education and mobilization, adherence support, and active case finding. They were also paid for their work and supported by the health centre staff at the clinic. A review of the programme suggests that community health workers with careful training and supervision are crucial in scaling up HIV treatment in the country (Rigodon *et al*. 2012).

Healthcare workers face a number of difficulties: heavy patient loads together with low morale, heightened fear of the risk of HIV infection, inadequate infrastructure, high staff turnover and other logistical problems, as has been found in other studies conducted in Africa (Buchan & Calman 2004; Cohen *et al*. 2009; Jaiantilal, Gutin, Cummings, Mbofana & Rose 2015). Until these constraints are removed, it will continue to thwart the full potential of healthcare workers to deal more effectively and holistically with patients in a high HIV prevalence setting. It is also likely to lead to the movement of healthcare workers out of public health facilities.
